# Can a toxin gene NAAT be used to predict toxin EIA and the severity of *Clostridium difficile* infection?

**DOI:** 10.1186/s13756-017-0283-z

**Published:** 2017-12-19

**Authors:** Mark I. Garvey, Craig W. Bradley, Martyn A. C. Wilkinson, Elisabeth Holden

**Affiliations:** 1 University Hospitals Birmingham NHS Foundation Trust, Queen Elizabeth Hospital Birmingham, B15 2WB, Edgbaston, Birmingham, UK; 2 Institute of Microbiology and Infection, The University of Birmingham, Edgbaston, Birmingham, UK

**Keywords:** *Clostridium Difficile*, NAAT, EIA, Severity, Mortality, *C. difficile* Infection

## Abstract

**Background:**

Diagnosis of *C. difficile* infection (CDI) is controversial because of the many laboratory methods available and their lack of ability to distinguish between carriage, mild or severe disease. Here we describe whether a low *C. difficile* toxin B nucleic acid amplification test (NAAT) cycle threshold (CT) can predict toxin EIA, CDI severity and mortality.

**Methods:**

A three-stage algorithm was employed for CDI testing, comprising a screening test for glutamate dehydrogenase (GDH), followed by a NAAT, then a toxin enzyme immunoassay (EIA). All diarrhoeal samples positive for GDH and NAAT between 2012 and 2016 were analysed. The performance of the NAAT CT value as a classifier of toxin EIA outcome was analysed using a ROC curve; patient mortality was compared to CTs and toxin EIA via linear regression models.

**Results:**

A CT value ≤26 was associated with ≥72% toxin EIA positivity; applying a logistic regression model we demonstrated an association between low CT values and toxin EIA positivity. A CT value of ≤26 was significantly associated (*p* = 0.0262) with increased one month mortality, severe cases of CDI or failure of first line treatment. The ROC curve probabilities demonstrated a CT cut off value of 26.6.

**Discussions:**

Here we demonstrate that a CT ≤26 indicates more severe CDI and is associated with higher mortality. Samples with a low CT value are often toxin EIA positive, questioning the need for this additional EIA test.

**Conclusions:**

A CT ≤26 could be used to assess the potential for severity of CDI and guide patient treatment.

## Introduction

*C. difficile* is an anaerobic, spore forming Gram positive bacillus found in diverse environments that is able to colonise and proliferate in the human gastrointestinal tract, especially following changes in the indigenous colonic microbiota after antibiotic use [1, 2]. *C. difficile* is one of the most common causes of healthcare associated infection and is responsible for 15 to 25% of all cases of antibiotic associated diarrhoea [2–4]. The infection causes an estimated 3000 deaths every year in the UK and 15,000–20,000 deaths in the USA, with associated case-fatality rates of 6–17% [4, 5]. It is associated with gut overgrowth of *C. difficile* and the production of toxins A, B, or both, which cause a range of effects, including gut mucosal damage, colitis, and pseudomembranous colitis [2, 4]. The primary mediators of inflammation in *C. difficile* infection (CDI) are large clostridial toxins, toxin A and toxin B. The toxins trigger a complex cascade of host cellular responses to cause diarrhoea, inflammation and tissue necrosis — the major symptoms of CDI [2, 4]. It must be noted a small number of healthy people naturally carry *C. difficile* in their large intestine and do not have ill effects from the infection [1].

Since *C. difficile* diarrhoea cannot be reliably distinguished from other causes of healthcare associated diarrhoea on clinical grounds alone, laboratory confirmation is essential [4, 6–10]. However, the diagnosis of CDI remains a contentious issue [4, 8, 9, 11]. For decades, toxin tests were favoured over culture for diagnosis of CDI because toxins mediate disease, detection was faster and this method provided evidence of toxin production in vivo that typically correlated better with clinical disease [3, 10, 12, 13]. Molecular tests such as nucleic acid amplification tests (NAAT) target toxin genes but are similar to culture in detecting *C. difficile* bacteria regardless of toxin production, making it unclear whether positive NAAT results reflect clinical disease [4, 12, 14]. A disadvantage for NAATs is that they detect toxin genes alone and not toxin production which is thought to be associated with CDI [15]. The uncertain clinical significance of positive NAAT results is problematic in inpatient healthcare facilities where *C. difficile* colonization is 5 to 10 times more common than CDI and non-infectious causes of diarrhoea [4, 12, 15]. A large multi-centre study in the UK led to a change in the Department of Health diagnostic guidance, after showing that an algorithmic approach was optimal for laboratory diagnosis [4]. Two stage algorithms have been widely adopted, and consist of; an initial screen using glutamate dehydrogenase (GDH) enzyme immunoassay (EIA) or toxin gene NAAT to detect the presence of *C. difficile*, followed by a second step which detects faecal toxin using either EIA or a cell cytotoxin assay [4, 8, 11, 13, 16, 17]. The cell cytotoxin assay is used to detect the presence of *C. difficile* toxin by its effect on human tissue cells grown in culture. This test is considered to be the ‘gold standard’ for diagnosing CDI, however it requires technical expertise and takes 24 to 48 h to produce a result [10].

In the UK, screen positive/toxin negative patients are usually regarded as being colonized with *C. difficile* rather than infected, based on a large multicentre prospective study showing that only patients with detectable faecal toxin had adverse outcomes [2–4]. However, outside the UK, such patients typically identified with NAATs as a screening test alone, are often regarded as having CDI [3, 18]. The role of *C. difficile* toxin gene NAAT for CDI diagnosis has largely been polarised between Europe and the US [3, 19]. Data from the National Healthcare Safety Network indicated in 2014 ~44% of US hospitals use NAAT for CDI diagnosis, whilst in Europe that level was much lower at 5% [3, 19]. Over diagnosis of CDI in hospitals using standalone NAAT can lead to unnecessary treatment causing clinicians to miss the real underlying diagnosis [3]. The outcome of patients who are NAAT positive/ toxin EIA negative has been shown to be indistinguishable from that of NAAT negative and toxin EIA negative patients (in terms of diarrhoea and CDI related complications) [3, 15]. Additionally, increased mortality is associated with the presence of faecal toxin but not with NAAT positive/ toxin negative sample results [15]. While qualitative results of NAAT have poor positive predictive value for CDI, could there be an alternative role for NAATs in CDI diagnosis? [12, 15] Here we aimed to determine if a low NAAT cycle threshold result can predict severity of CDI and/or mortality using data from our institution over the past 5 years.

## Materials and methods

### Setting

Queen Elizabeth Hospital Birmingham (QEHB), part of University Hospitals Birmingham (UHB) NHS Foundation Trust is a tertiary referral National Health Service teaching hospital in Birmingham, UK that provides clinical services to over one million patients every year.

### *C. difficile* Testing

In line with national guidance, an algorithmic approach to identifying CDI is undertaken at QEHB [4, 20, 21]. A three-stage algorithm is employed. Briefly, any patient with ≥1 episode of unexplained diarrhoea had their faecal specimen tested for CDI. The CDI testing algorithm consists of an initial screening step using a Premier GDH EIA (Meridian Bioscience, Cincinnati, Ohio), followed by a NAAT (Cepheid, Xpert™ *C. difficile*, US) for GDH positive samples only. The premier GDH involved undertaking an enzyme immunoassay looking for the presence of GDH as previously descrbed [4, 21]. The Cepheid NAAT is a real-time PCR assay targeting the toxin genes (B toxin gene and *tcdB*) [12, 14]. The Cepheid PCR targets the toxin B gene (t*cdB*) as it is independently capable of causing CDI [12, 14]. The cycle threshold (CT) value of the Cepheid real time PCR describes the number of cycles needed until DNA amplification occurs exponentially in a real-time PCR assay and they are correlated with the amount of target sequence in the sample [12]. All samples which were GDH and NAAT positive have a Premier Toxins A and B EIA (Meridian Bioscience, Cincinnati, Ohio) which is undertaken once a week [20, 22]. The Premier Toxins A and B EIA was undertaken as previously described, with a sensitivity of 94.7% and specificity 97.3% quoted by the manufacturers when compared to the reference cytotoxin method [4, 21].

### Study design and definitions

All diarrhoeal samples (Bristol stool type 5–7) [23] from patients between Jan 2012 to Dec 2016 at QEHB positive by GDH and NAAT were included in the study which equated to 1346 patients.

### CDI episode

At QEHB a CDI episode was defined as the presence of a positive test result for toxigenic *C. difficile* by GDH and NAAT and the presence of unexplained diarrhoea (≥1 episode of unexplained diarrhoea). Severity of CDI was based on the Public Health England (PHE) toolkit for management of *C. difficile* [20]. A severe *C. difficile* case was defined as: WCC >15 × 10 [9] g/dL; acutely rising blood creatinine (e.g. >50% increase above baseline); temperature > 38.5 °C; or evidence of severe colitis (abdominal signs, radiology) [20].

### Recurrent CDI and treatment failure

Recurrent CDI was defined as the return of diarrhoea (≥1 episode of unexplained diarrhoea) within 30 days of a previous CDI episode and the presence of a positive test result for toxigenic *C. difficile* by GDH and NAAT [20]. Treatment failure was defined as cases where failure to respond to treatment resulted in a change of management of the patient [12].

### Clinical data collection

Patient data collected at the time of a positive result included: patient demographics (age, sex), markers for CDI severity (white cell count, C-reactive protein, serum creatinine, serum albumin, temperature, stool frequency) and mortality (one month and 3-month all-cause mortality). Clinical severity data were obtained from 80 patients with the lowest and 138 patients with the highest CT values; mortality data was collected from all 1346 patients in the study.

### Statistical analysis

Cepheid NAAT CT values were compared to Premier Toxins A and B EIA positivity and patient mortality (one and three month). All analyses including receiver operating characteristic curve (ROC), Youden’s index, error rates and univariate logistic regression models were performed using the hmeasure, plotROC and base packages in the statistical programming language R [24–26].

### ROC curve

The performance of NAAT CT value as a classifier of toxin EIA positivity was assessed using a ROC curve and the area under the ROC curve (AUC). The sensitivity (TPF; True Positive Fraction), specificity, False Positive Fraction (FPF), Positive Predictive Value (PPV) and negative predictive value (NPV) of the prediction rule of low CT values corresponding to EIA positivity were calculated using the hmeasure and plotROC packages in R [24, 25]. To determine a NAAT CT cut off value, two commonly-used strategies were used in the study picking a threshold that minimises the error rate and picking one that maximises Youden’s index [27, 28].

### Linear regression mortality

Logistic regression models were used to assess NAAT CT values as an explanatory variable for mortality and EIA toxin positivity. These models were fitted using R [26].

## Results

### Samples

**Fig. 1:**
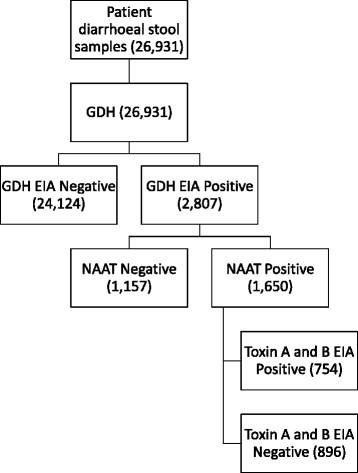
Algorithm of CDI testing at QEHB with the number of results obtained between Jan 01 2012 and Dec 31 2016 depicted

Between Jan 01 2012 and Dec 31 2016; 26,931 diarrhoeal stool samples were tested for CDI at QEHB. Of the 26,931 samples 2807 (10%) were GDH positive, of these 1650 (59%) were NAAT positive. Of the 1650 GDH and NAAT positives, 754 (46%) were toxin EIA positive (Fig. 1).

### NAAT CT vs EIA

**Fig. 2:**
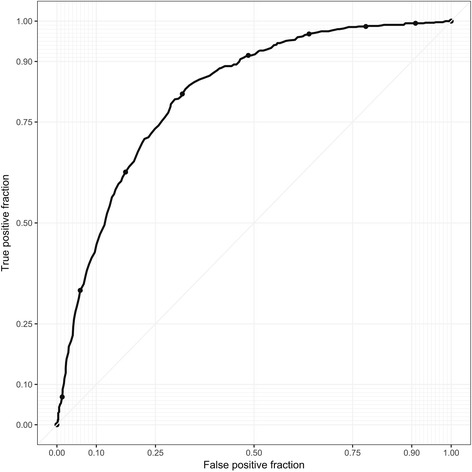
ROC curve comparing NAAT CT value vs toxin EIA positivity. Note: The area under the ROC curve (AUC) was equal to AUC = 0.819. The *y*-axis is the True Positive Fraction, or sensitivity, and the *x*-axis is the False Positive Fraction, which is equal to 1 − specificity. The True Positive Fraction is the probability that for a fixed cut off, *c*, the classifier (*M*) gives a positive result when the true result (*D*) is positive, i.e. TPF(*c*) = *P*(*M* ≥ *c*| *D* = *Positive*). In a similar fashion, the False Positive Fraction is the probability that for a fixed cut off the classifier gives a positive result when the true result is negative, i.e. TPF(*c*) = *P*(*M* ≥ *c*| *D* = *Negative*)

The mean toxin B CT-value of all samples was 27.1 (*n* = 1650). Low CT values were associated with toxin EIA positivity (Table 1). A CT value of <26 was associated with ≥72% toxin EIA positivity as compared to a CT value ≥26 where a 23% EIA positivity rate was observed (Table 1). To test the model of low CT values corresponding to EIA positivity a ROC curve was calculated (Fig. 2). The area under the ROC curve (AUC) was equal to AUC = 0.819 (Table 2). For our data, we have a minimum error rate of approximately 0.251 which occurs at toxin thresholds 26.6 and 26.7, whilst the maximum value for Youden’s index is approximately 0.507, which occurs at the toxin threshold 26.7. A cut off CT value ≤26 was chosen as a value likely to yield EIA positivity, whilst minimising the risk of over diagnosis (Table 2).

**Table 1:** The number of toxin EIA samples against a selection of NAAT CT value ranges with patient mortality within 1 month and 3 months

CT Value	EIA Toxin Positive ^a^	Total EIA ^b^	Mortality/month ^c^	Mortality/3 months ^c^
18	1 (100%)	1	1	1
19	4 (80%)	5	2	2
20	32 (84%)	38	7	12
21	80 (85%)	94	17	25
22	113 (80%)	140	16	29
23	119 (72%)	166	23	31
24	106 (67%)	157	27	39
25	96 (56%)	171	21	34
26	63 (50%)	125	12	19
27	52 (37%)	139	19	34
28	24 (27%)	89	7	13
29	23 (24%)	95	10	16
30	17 (20%)	83	8	16
31–37	24 (7%)	347	41	60
All	754 (46%)	1650	210	331
18–25	551 (72%)	772	113	173
26–37	203 (23%)	878	97	158

^a^ All samples are GDH, NAAT and toxin A and B EIA positive

^b^ Total number of toxin A and B EIAs undertaken from GDH and NAAT positive samples including both negative and positive results for toxin A and B

^c^ Reflects the number of patients with 1 month or 3-month mortality

**Table 2:** Comparison of NAAT CT values (1/*c*) vs toxin EIA positivity

CT value	Sensitivity (TPF)	Specificity	FPF	PPV	NPV
20	0.008	0.998	0.002	0.750	0.544
21	0.050	0.992	0.008	0.844	0.554
22	0.163	0.975	0.025	0.848	0.581
23	0.314	0.944	0.056	0.826	0.621
24	0.472	0.891	0.109	0.784	0.667
25	0.613	0.834	0.166	0.756	0.719
26	0.733	0.751	0.249	0.713	0.770
27	0.820	0.682	0.318	0.684	0.818
28	0.883	0.585	0.415	0.642	0.856
29	0.915	0.507	0.493	0.610	0.876
30	0.948	0.426	0.574	0.582	0.907
35	0.995	0.076	0.924	0.475	0.944

The table shows CT values against various diagnostic measures used in the ROC analysis including sensitivity and specificity. The area under the ROC curve (AUC) was equal to AUC = 0.819, corresponding to a CT value of ≤26

*TPF* True Positive Fraction, *FPF* False Positive Fraction, *PPV* Positive Predictive Value, *NPV* Negative Predictive Value

To investigate whether NAAT CT value correlates with toxin EIA positivity, the following logistic regression model was used: Y = 9.50654–0.36306X (where the response variable, *Y*, is the logit function of the probability of a positive result from the toxin EIA, and *X* is the NAAT CT value). The *p* value for the regression coefficient for the explanatory variable is *p* < 2 × 10^−16^, demonstrating that the CT value there is inversely proportional to the probability of a positive result from the toxin EIA. The model predicts the approximate probabilities of a positive EIA result as 0.904, 0.606, 0.517, 0.200 and 0.039 for the CT values 20, 25, 26, 30 and 35, respectively.

### NAAT CT vs mortality

Between 2012 and 2016, 210 patients died within a month of a *C. difficile* NAAT positive result, this increased to 331 after 3 months (Table 1); 805 patients did not die. A logistic regression model was used to identify whether CT value corresponds with mortality. When looking at the number of deaths within 1 month, the following regression model was used: *Y* =  − 0.85203 − 0.03996*X* (the response variable, *Y*, is the logit function of 1-month mortality, and the explanatory variable, *X*, is the NAAT CT value). The *p* value for the regression coefficient for the explanatory variable is *p* = 0.0262, indicating that a low CT value is associated with mortality. The model predicts the approximate probabilities of death within 1 month as 0.161, 0.136, 0.114 and 0.095 for the CT values 20, 25, 30 and 35, respectively.

### NAAT CT vs severity of CDI

Clinical data were obtained from 80 patients with the lowest and 138 patients with the highest CTs. 99 out of 138 (72%) patients with a CT value between 18 and 21 had severe/recurrent CDI. Failure of first line treatment with metronidazole was observed in 23 out of the 39 cases with low CT values that had been classified as mild CDI, resulting in escalation to second line therapy such as vancomycin, fidaxomicin or faecal microbiota therapy. Of the 80 patients with CT values between 35 and 37, 74 (92%) of the patients had mild CDI with resolution of symptoms following first line therapy of metronidazole.

## Discussion

In our patient population, analysis of all *C. difficile* NAAT positive stool samples between 2012 and 2016 indicated that a low NAAT CT value was independently associated with toxin EIA positivity, higher mortality and CDI severity. We propose that a microbiological marker cut off, based on CT value (≤26), could be used as a predictor of poor outcome and serve as a severity indicator to guide treatment of CDI.

In this study, regression analysis demonstrated that low CT values were associated with toxin EIA positivity. With a CT value ≤26 we saw ≥72% toxin EIA positivity; applying a logistic regression model we demonstrated an association between low CT values and toxin EIA positivity. Conversely a CT value of ≤26 would miss 28% of patients that were EIA positive. It is not surprising that a low CT value is linked with toxin EIA positivity as previous studies have suggested expression of toxin is associated with the severity of CDI, and a correlation between faecal toxin levels and *C. difficile* counts has been reported in mice [12]. Dionne et al.*,* previously identified that amplification CT values correlate to bacterial load [29]. They demonstrated toxin positive samples had higher bacterial loads than toxin negative samples and there was a significant inverse correlation between PCR CT values and bacterial loads [29]. In the present study, low CT values inversely correlate to toxin assay positivity, one could imply that CT values of toxin B genes act as a marker of the amount of toxin produced [12]. However, investigations seeking to link quantitative toxin production with *C. difficile* virulence have not been conclusive [12]. Further work is warranted to explore this.

In the UK, two stage algorithms for CDI have been adopted [4, 10]. Often a GDH test for screening is employed followed by a toxin EIA [4, 10]. Eyre et al.*,* (2017) sequenced all GDH positive patients and compared the probability of faecal toxin positive and toxin negative patients being likely sources of transmission, that is, having *C. difficile* genetically linked to a subsequent isolate in another patient [30]. They concluded faecal toxin negative patients were similarly infectious to faecal toxin positive patients; faecal toxin status did not affect transmission rates [30]. They concluded strategies to identify and institute infection control measures around patients with potentially toxigenic *C. difficile* without detected faecal toxin are likely to reduce CDI incidence [30]. NAAT could be used as a screening test as it is rapid and highly sensitive to aid detection of such cases from an infection control perspective [31]. In addition with concerns about the lower sensitivity of toxin EIA testing increased usage of NAAT in hospitals worldwide has been seen [12]. The results in our study suggest a NAAT CT value of ≤26 corresponds to toxin EIA positivity. In our setting, a GDH test followed by NAAT using the CT value as marker of EIA positivity would seem to be sufficient to pick up the majority of EIA positive cases. NAAT detects the DNA of the toxin gene of *C. difficile* rather than the presence of toxin in stool samples, however in our study a low CT value shows a strong association with toxin EIA positivity. Our finding is in line with the observations of Jazmati et al.*,* (2016) that free toxin detection is associated with low CT-values [32]. As in our study Jazmati et al.*,* (2016) showed CT-values were significantly lower in specimens that were positive in toxigenic culture (26.2 ± 4.5, *n* = 100 versus 30.5 ± 5.0, *n* = 65; *p* < 0.001) [32]. Senchyna et al.*,* (2017) also found low CT values predicted free toxin results; a CT value of <26.35 could sensitively predict 96.0% of toxin EIA / PCR stool samples with a negative predictive value of 97.1% and with a specificity of 78.0% using both EIA toxin and cell cytotoxin assay [33]. In our study it is important to note that the toxin EIA has a sensitivity of 94.7% which could affect the CT value of ≤26 corresponding to toxin EIA positivity. One main disadvantage of NAATs is that they do not detect the presence of biologically active toxin in stool specimens [15]. Toxins expressed by *C. difficile* are the main virulence factor, and some feel that the presence of toxin in stool is a positive correlation of disease [4, 12, 15]. The significance of detecting *C. difficile* in the absence of the toxins, such as in the patient who tests positive by NAAT but negative by toxin EIA, is unclear [15].

The majority of large studies on *C. difficile* diagnostics show toxin EIA is the best predictor of outcome, with NAAT tests providing no additional information about disease severity [4, 9, 11, 13]. However, in our study 72% (*n* = 99/138) patients with a CT value between 18 and 21 had severe/recurrent CDI based on PHE guidelines for management of CDI [20]. In the mild cases, with a CT value between 18 and 21, we often saw treatment failure with our first line therapy of metronidazole requiring escalation of treatment. In contrast, 92% (*n* = 74/80) of the patients with a high CT value had mild CDI and responded to treatment. In our setting, the CT value could be used to guide treatment of patients with CDI. It must be noted we have only looked at a small number of patients with either the lowest or highest CT values. Reigadas et al.*,* (2016) also showed patients with severe disease had significantly lower CT-values (toxin B CT value of <23.5) compared with patients in the other groups [12]. In our study, we also looked at the mortality within a month of CDI. It must be noted we have looked at all-cause mortality and not mortality specifically attributed to CDI. However, a significant association between low CT value and mortality was observed. Rao et al. (2015) found that there was no correlation between amplification CT values and severe CDI or all-cause mortality, however in our study; the mean toxin B amplification CT value was 27.1, which was much lower than that reported by Rao et al. (34.3) [34]. There were limitations in the study by Rao et al.*,* (2015) which included sampling amounts and specimen transport. [34]

It is often difficult to distinguish between healthcare associated diarrhoea and CDI [1]. In CDI, the spectrum of clinical disease ranges from mild diarrhoea to toxic megacolon, colonic perforation and death [1]. Research suggests that both strain characteristics and the host’s immune response influence CDI severity, recurrence risk and mortality [1]. Jazmati et al.*,* Reigadas et al.*,* and Senchyna et al.*,* all showed a correlation between low NAAT CT values and severity and/or patient outcome [12, 32, 33]. To our knowledge, this is the largest study reporting a significant association between CT values of a NAAT *C. difficile* test vs toxin EIA positivity and mortality. There is growing evidence that NAAT CT values could be used to predict CDI severity, recurrence risk and mortality.

## Conclusion

Using ROC analysis to identify a relationship between CT value and EIA toxin positivity we were able to predict a cut off CT value threshold of ≤26 which was associated with a high probability of EIA positivity. In addition, we showed a low CT value was associated with mortality within a month. Low CT values were also associated with more severe cases of CDI in our setting. A CT value of ≤26 could be used as an adjunct in the testing algorithm for *C. difficile* to enable detection of toxin EIA positive strains, as well as be used in conjunction with clinical parameters to assess disease severity and guide treatment of patients with CDI. However, we recognise that more work is needed to understand the nature of any link between low CT values and severity of CDI. We also suggest that, since a low CT value correlates with a positive toxin EIA, a CT ≤26 could in theory be used to guide reporting and streamline the contentious testing issues of CDI.

Finally, we hypothesise that increased presence of *C. difficile* in the gastrointestinal tract of affected individuals leads to enhanced toxin detection, resulting in lower CT values and reflecting the phenomenon of gut dysbiosis that is thought to precipitate this disease.
